# Health economic evaluation of left atrial appendage closure in patients with atrial fibrillation and at high risk for stroke and bleeding: a study protocol

**DOI:** 10.3389/frhs.2026.1771683

**Published:** 2026-07-01

**Authors:** Franziska Claus, Marco Müller, Eric Faß, Joanna Diesing, Carsten Skurk, Claudio Seppelt, Denitsa Meteva, Andi Rroku, Ingo Eitel, Leif-Hendrik Boldt, Ulf Landmesser, Eberhard Wille, Ines Weinhold

**Affiliations:** 1 Scientific Institute for Health Economics and Health System Research, Leipzig, Germany; 2 Department of Cardiology, Angiology and Intensive Care Medicine, Deutsches Herzzentrum der Charité, Charité-University Medicine Berlin, Campus Benjamin Franklin, Berlin, Germany; 3 German Center for Cardiovascular Research (DZHK), partner site Berlin, Berlin, Germany; 4 German Center for Cardiovascular Research (DZHK), partner site Hamburg/Kiel/Lübeck, Lübeck, Germany; 5 University Heart Center Lübeck, Medical Clinic II (Cardiology/Angiology/Intensive Care Medicine), Lübeck, Germany; 6 Department of Cardiology, Angiology and Intensive Care Medicine, Deutsches Herzzentrum der Charité, Charité-University Medicine Berlin, Campus Virchow, Berlin, Germany; 7 Department of Economics, University of Mannheim, Mannheim, Germany

**Keywords:** atrial fibrillation, cost-effectiveness, cost–utility analysis, health economic evaluation, percutaneous catheter-based left atrial appendage closure

## Abstract

**Background:**

Atrial fibrillation (AF) is the most common cardiac arrhythmia. The associated risk of stroke can be effectively reduced by oral anticoagulation; however, these increase bleeding risk or can even be contraindicated. Clinical studies indicate that percutaneous catheter-based left atrial appendage closure (LAAC) may represent an effective alternative for stroke prevention in this high-risk patient group. While the clinical and economic benefits of LAAC have been compared with standard drug therapy indirectly, direct comparisons are lacking, especially for patients with AF at high risk of stroke and bleeding. EvaClosure, a substudy of the CLOSURE-AF-DZHK16 trial, was designed to describe current therapy paths of patients with AF and high risk of stroke and bleeding, while quantifying resource utilization and healthcare costs. Moreover, this study aims to evaluate the health economic impact of LAAC in patients with high risk of stroke and bleeding, compared with the best physician-directed medical therapy (including direct oral anticoagulants when eligible).

**Methods:**

Data gathered in CLOSURE-AF address the effect of different treatment options on health-related quality of life; analyses of resource utilization and healthcare costs are based on claims data from a German statutory health insurance (SHI) provider. Economic information derived from SHI data is complemented by a validated resource use measurement instrument capturing non-medical and indirect costs. The cost–utility analysis uses a Markov model, for which parameters are quantified from statistically matched SHI claims and CLOSURE-AF clinical study data, supplemented by data from the literature.

**Discussion:**

The combination of these databases allows a comprehensive, valid, and policy-relevant assessment of costs and benefits related to LAAC. Moreover, the advantages of the different databases are used for health economic evaluation. The results of this study are expected to generate robust evidence to inform health economic evaluations and provide valuable insights for SHI providers and policymakers on the societal and economic impact of LAAC.

**Clinical Trial Registration:**

ClinicalTrials.gov, Identifier NCT03463317. The clinical study CLOSURE-AF-DZHK16, including the substudy EvaClosure, was registered at ClinicalTrials.gov PRS (Identifier: NCT03463317, Posted: March 13, 2018, Update: July 30, 2021).

## 1 Introduction

### 1.1 Background

Atrial fibrillation (AF) is the most common cardiac arrhythmia, with the current rate of prevalence ranging between 2% and 4% (1); the prevalence increased in the last 20 years and further augmentations are expected (2, 3). For Germany, current studies have shown a prevalence rate of 3.1% and a significant increase in recent years due to demographic change and a growing awareness of this disease (4, 5). The current estimated lifetime risk of AF at age 55 is 37%, which has also grown in recent years (6, 7). AF increases stroke risk about five-fold, with 15% to 25% of all strokes attributed to AF (8, 9). AF is also associated with a higher risk of mortality (10). In addition to the clinical outcomes, AF causes high economic burden for healthcare systems and society (11, 12), whereby acute care costs of complications contribute substantially to this burden (13). The latest available estimates suggest an economic burden of up to 3.4 billion euros for Germany, primarily due to hospitalizations (14). Because of the increasing prevalence of AF, the associated costs are expected to grow as well (15).

Although non-vitamin K antagonist oral anticoagulants (NOACs) substantially reduce the risk of stroke and systemic embolism in patients with AF, their use in patients with high bleeding risk may be limited by safety concerns and long-term treatment persistence. In pivotal phase III trials and subsequent meta-analyses, the annual rate of incidence of major bleeding during NOAC therapy ranged approximately from 2% to 4%, depending on patient risk profile and drug regimen, with gastrointestinal bleeding representing the most frequent severe bleeding manifestation (16–19). Furthermore, contemporary registry data indicate that persistence with oral anticoagulation declines progressively over time, with discontinuation or non-adherence rates of approximately 15%–30% within the first year of treatment and substantially higher cumulative discontinuation rates during longer follow-up times. The reported reasons include bleeding complications, perceived treatment burden, multimorbidity, drug–drug interactions, and patient preference (10, 20). Consequently, a considerable proportion of patients with AF and high bleeding risk remain undertreated or untreated in routine clinical practice. Current European Society of Cardiology guidelines therefore emphasize the need for individualized stroke prevention strategies and recommend a consideration of left atrial appendage closure in selected patients with contraindications to long-term anticoagulation (21).

Preliminary evidence suggests that percutaneous catheter-based left atrial appendage closure (LAAC) may represent an effective alternative for stroke prevention and may eliminate the need for permanent anticoagulation, especially for patients in this high-risk group (22). However, current research lacks a direct comparison of LAAC with NOAC therapy at a larger scale since this high-risk patient group is often not represented comprehensively in clinical studies. Therefore, additional evidence based on comparative studies is needed to recommend LAAC for routine clinical practice (23).

The CLOSURE-AF-DZHK16 study addresses this research gap. CLOSURE-AF is a prospective, randomized clinical trial involving 42 study centers in Germany^1^ that analyzes the clinical benefit of LAAC in patients with non-valvular AF (NVAF) at high risk of stroke and bleeding (high-risk group)^2^ compared with best physician–directed medical care (including anticoagulation, when tolerated). Patient enrollment began in February 2018, with a study completion date in November 2024. Subjects were randomized 1:1 in the intervention and control group, receiving either catheter-interventional LAAC with a CE-certified LAA occluder (e.g., Watchman™, Amplatzer Amulet) or physician-directed best medical care [NOACs or vitamin K antagonists (VKA), antiplatelet therapy, no antithrombotic/antiplatelet therapy in case of contraindications] chosen at the discretion of the treating physician (23).

Changing standard care by introducing a novel medical device must be supported by evidence of both clinical and economic benefits. However, evidence on LAAC cost-effectiveness in high-risk patients is lacking. Therefore, an incremental analysis of the costs and benefits of the intervention compared with standard medical therapy is essential to adequately reflect its utility for patients as well as the economic impact from the perspectives of both SHI and society. Previous health economic evaluations of LAAC (25–32) predominantly considered patients with NVAF without accounting for contraindications to (N)OACs. All but one of the eight studies evaluated LAAC cost-effectiveness based on incremental costs per quality-adjusted life year (QALY). Two out of the eight identified studies considered NVAF patients with contraindications, for which the results suggest that, depending on the time horizon, LAAC is cost-effective compared with therapy with apixaban or aspirin or represents the dominant treatment alternative for patients with AF at risk of stroke and contraindications to (N)OACs (30, 31). Based on health economic modeling, Reddy et al. (30) found that LAAC was more clinically effective and, after a time horizon of eight years, also more cost-effective in this high-risk group. However, none of the aforementioned studies compared LAAC treatment with a control group, where treatments are not limited to those specific medications. Such a restriction does not reflect real-world standard care, which is relevant for economic evaluation from the perspectives of both SHI and society. Moreover, in Germany, no health economic evaluation has yet been conducted for the—potentially particularly effective—clinical use of LAAC in this specific high-risk population recruited in the CLOSURE-AF trial.

### 1.2 Objective

In parallel to the CLOSURE-AF study, EvaClosure^3^ adds to previous evidence by comparing the LAAC procedure with best medical therapy prescribed by practitioners in a high-risk patient pool (including patients with potential contraindications to NOACs), evaluating its real-world health economic impact. EvaClosure uses both primary clinical study data and real-world German SHI claims data to analyze LAAC cost-effectiveness in comparison with currently available pharmaceutical alternatives. The incremental cost-effectiveness ratio (ICER) of the LAAC treatment is calculated using data from the different databases. A Markov model is used to further evaluate the health economic effects in a lifetime horizon. The model is parameterized with health-related quality-of-life effects (aggregated to QALYs) in the different study arms of the CLOSURE-AF study as well as direct and indirect costs estimated from SHI claims data, supplemented with patient-reported healthcare resource use and data from the literature.

In summary, EvaClosure aims to

- assess resource utilization and medical costs associated with a high risk of stroke and bleeding in patients with AF
- assess direct and indirect costs in high-risk AF patients and of LAAC intervention vs. best medical care
- assess the utility effects (QALYs) of LAAC compared with best medical care in high-risk AF patients
- evaluate the cost-effectiveness of the LAAC intervention compared with best medical care based on the ICER two and five years after intervention as well as in a life-time-horizon from the perspectives of both SHI and society.

## 2 Methods

### 2.1 Study design

EvaClosure is a longitudinal cohort study of adults with AF and high risk of stroke and bleeding, based on two independent study populations: an SHI claims data population and the EvaClosure population—a subpopulation of patients recruited in the CLOSURE-AF trial. There is no patient-individual linkage of the CLOSURE-AF trial data with the SHI claims data; however, since information from both data sources converge in the health economic evaluation, statistical matching is used to establish maximum similarity between both study populations.

Pseudonymized CLOSURE-AF trial data are used for matching, including study participants' demographic information (e.g., gender, age), medical history and comorbidities, therapy (treatment arm, medications), the CHA_2_DS_2_VASc and HAS-BLED scores, as well as periprocedural events, complications, and adverse events. A matched sample is drawn from the eligible SHI population, for which the utilization of healthcare services and corresponding costs are determined. The basis of data for the health economic evaluation and Markov modeling is supplemented by primary data on quality of life collected using the EQ-5D-5L instrument (33, 34) aggregated to QALYs, as well as information on further health resource usage captured by the validated and standardized FIMA^4^ questionnaire (35, 36) [modified version, see the section “Outcome measures and analyses” (A2) for details on the modifications]. The survey-based data are collected within the CLOSURE-AF trial every six months up to two years after patient inclusion.

### 2.2 EvaClosure primary data

#### 2.2.1 EvaClosure study population

The EvaClosure population comprises a subgroup of the CLOSURE-AF trial participants who consented to participate in the health economic evaluation (=EvaClosure study). Thus, the EvaClosure population includes both newly recruited patients and study participants who had already been enrolled in the CLOSURE-AF trial (see the section “trial data collection” for further details).

Patients enrolled in the CLOSURE-AF study do not have to meet any further criteria (apart from giving consent) to participate in the EvaClosure substudy. The eligibility criteria for CLOSURE-AF have been described elsewhere in detail (23); the trial includes patients 18 years or older with documented AF (paroxysmal, persistent, or permanent) and with high risk of bleeding or stroke. High risk of bleeding and stroke is determined by the CHA_2_DS_2_VASc and HAS-BLED scores as well as other characteristics [see (37, 38)]. Moreover, patients must be suitable candidates for catheter-interventional atrial appendage closure. Only those patients with an absolute contraindication to ASA and other antiplatelet drugs such as P2Y12-inhibitors like clopidogrel or with other comorbidities requiring long-term (N)OAC treatment were excluded from the study. Patients in the control group will be treated at the discretion of their local physician according to recommendations based on their specific bleeding risk. Patients will receive one of four approved NOAC treatments in different doses, including the relevant dose-reduction criteria: dabigatran, rivaroxaban, apixaban, or edoxaban. Table 1 summarizes the approved (N)OACs and doses. In case of contraindications to NOACs, patients may be either treated with vitamin-K antagonists (warfarin, phenprocoumon) and antiplatelet agents or there will be no stroke preventive treatment.

**Table 1:** (N)OACs and approved/studied doses across indications.

(N)OACs	Higher dose	Lower dose
Dabigatran	150 mg BID (CrCl >30 mL/min)	110 mg BID [Age >80, Interacting drugs (e.g., verapamil), CrCl 15–30 mL/min]
Rivaroxaban	20 mg/d (CrCl >50 mL/min)	15 mg/d (CrCl 15-50 mL/min)
Apixaban	5 mg BID	2.5 mg BID (if two of the following present: age >80, weight <60 kg, serum creatinine >1.5 mg/dL)
Edoxaban	60 mg/d	30 mg/d (weight <60 kg, CrCl 15-50 mL/min)

BID = twice daily; CrCl, creatinine clearance.

#### 2.2.2 EvaClosure sample size and recruitment

Within the health economic evaluation, the LAAC effect on health-related quality of life compared with standard therapy will be measured in terms of utilities; considering the duration of the underlying health conditions, these will be aggregated to QALYs. A two-sided, unpaired t-test (or Welch test) must be performed to ensure that differences in QALYs found between groups are not random. According to Freeman et al. (26), the utility value for the NOAC dabigatran is 0.994 and the 10% percentile is given as 0.975. Assuming a normal distribution, this results in a standard deviation of 0.015. In the same reference, the utility value for LAAC is reported as 0.998, while the 10% percentile of 0.994 results in a standard deviation of 0.003. In combination, assuming that both intervention and control groups are equally powerful, this results in an effect size of 0.37. With a targeted power of 85% and a significance level of 5%, this results in a case number of 264 patients (or 132 per group). Assuming a loss-to-follow-up-rate of 10%, the required sample size is 294 patients (147 per group). Recruitment for EvaClosure started in January 2021 and was completed in September 2023 when the target number of cases was reached. After enrollment, patients were followed up over a period of two years, with the data collection exercise completed in November 2024.

#### 2.2.3 Trial data collection

Data collection on health-related quality of life within CLOSURE-AF began in March 2018 and ended in November 2024. Data on the use of health resources based on a modified version of the FIMA questionnaire^5^ were collected by standardized interviews conducted by a trained study nurse. Data were collected every 6 months and intended to cover a two-year period starting from baseline. According to the CLOSURE-AF study visit plan, patients were asked to complete the FIMA interview at baseline as well as at visit number V5–V8 (see Supplementary Table S2 in Supplementary Appendix A). Up to five observation points were planned for each patient, including baseline. Because the EvaClosure substudy started later than the main clinical trial, informed consent for some patients was obtained retrospectively starting from January 2021. For the retrospectively enrolled EvaClosure participants, the FIMA was used at the first possible visit. As a result, for patients who consented to EvaClosure at V8, that is 24 months after inclusion in the clinical trial, there was only one measurement point. For consecutively enrolled patients, a two-year follow-up with five measurement points was scheduled. An overview of the visit schedule and the respective data collected is provided in Supplementary Table S2 in Supplementary Appendix A.

### 2.3 SHI claims data

#### 2.3.1 SHI study population

The SHI population for EvaClosure is selected from the data pool of a German statutory health insurance fund that comprises over 3 million insured persons. EvaClosure uses data from the years 2015 to 2021. Patients are selected if they

- are 18 years or older, and
- have a diagnosis code (of the 10th revision of the International Statistical Classification of Diseases and Related Health Problems; ICD-10) for AF documented in at least two different quarters during the baseline period (I48.0, I48.1, I48.2, I48.9)

Patients are excluded from the sample in case of

- discontinuous data coverage (e.g., due to interrupted insurance periods or change of health insurance fund)
- an absolute contraindication for ASA (an ICD-10 code for gastric or duodenal ulcers (K25.- or K26.-) or a prescription for methotrexate [Codes within the Anatomical Therapeutic Chemical Classification System (ATC): L01BA01 or L04AX03])
- previous heart transplant (code within the German Operation and Procedure Classification System; OPS: 5–375)
- malignant neoplasm of the heart, mediastinum, and pleura (ICD: C38.-) or
- pregnancy, birth, or postpartum diagnosis in the baseline period (ICD: O–.—)

Patients are assigned to the high-risk group if in the baseline period they additionally have the following characteristics:

- CHA_2_DS_2_VASc-Score ≥ 2, and
- high risk for bleeding, i.e., if at least one of the following criteria are met:
  ○ HAS-BLED value of ≥ 3, or
  ○ an ICD-10 code for other non-traumatic intracranial hemorrhage (I62.-), or
  ○ gastrointestinal bleeding (K92.2), or
  ○ hematuria (R31), or
  ○ bleeding from the respiratory tract (R04.-), or
  ○ chronic kidney disease (N18.4)

From the high-risk patients, the LAAC intervention group is selected using the following inclusion criteria:

- Diagnosis-related group (DRG): F95A (interventional septal occlusion, age < 18 years, or atrial appendage occlusion) and
- OPS code 8-837.s0 (since 2016) or 8-837.s (before 2016)

The control group (best medical care group) is also selected from the group of high-risk patients, defined by the following criteria:

- no LAAC [DRG: F95A; OPS code 8-837.s0 (since 2016) or 8-837.s (before 2016)] procedure documented and
- treatment with best medical care (NOACs (dabigatran (ATC code B01AE07), rivaroxaban (ATC code B01AF01), apixaban (ATC code B01AF02), edoxaban (ATC code B01AF03)) or VKA [phenprocoumon (ATC code B01AA04), and warfarin (ATC Code B01AA03)]

For both groups, an additional exclusion criterion according to the CLOSURE-AF trial of a severe renal failure (ICD N18.5) or current requirement for dialysis (ICD Z49.1, Z49.2 in the quarter of inclusion) is applied (23).

#### 2.3.2 SHI data collection

SHI patient data are transferred for each year for the time period from 01.01.2015 to 31.12.2021; for each patient included, at least one year of baseline data (used to confirm inclusion/exclusion criteria prior to observation period) and at least two years of follow-up data are captured. The data include information on

- sociodemographic characteristics (age, gender, and insurance period)
- outpatient medical care [diagnoses (ICD-10), quarter of diagnoses, procedures, costs of care]
- pharmaceuticals (dispensed drugs, numbers of packages, prescription date, dispense date, and costs of therapy)
- medical devices and allied health services (type of therapy or device, service provider, and therapy duration and costs)
- unemployability (diagnoses, sick leave durations and payments, and durations), and
- inpatient medical care: main and secondary diagnoses (ICD-10) upon discharge, surgeries and procedures (OPS), date and cause of admission, date and reason for discharge, and DRG code.

### 2.4 Outcome measures and analyses

The EvaClosure study design consists of four modules that build on one another (see Table 2). For module A1, a cost-of-illness study assesses direct and indirect costs associated with a high risk of stroke and bleeding in patients with AF. Focusing on the high-risk group only, module A2 compares direct and indirect costs associated with LAAC treatment to best medical care. Module B1 quantifies the utility effect of LAAC in terms of QALYs gained compared with standard of care via data of the EvaClosure study population. The subsequent module B2, on the other hand, combines SHI claims data and data from the EvaClosure study population as well as CLOSURE-AF trial results to conduct a cost–utility analysis.

**Table 2:** Overview study modules, design, and outcome measures.

Module	Main Objectives	Study population	Design	Time frame	Costs/Outcomes
A1	To assess cost of illness to high-risk of stroke and bleeding in patients with AF	SHI study population, divided into the high-risk AF group and the AF group without high risk	Longitudinal cohort study, retrospective (difference-in-differences)	2015–2021; 1 year before and 4 years after inclusion per individual	direct and indirect costs
A2	To assess direct and indirect costs of LAAC intervention vs. best available medical care in high-risk patients with AF	EvaClosure study population: high-risk AF patients divided into intervention and control groups as per the CLOSURE-AF protocol.	Longitudinal cohort study, prospective (difference-in-differences)	2018–2025; enrollment starting in 01/2021 with a follow-up of up to 24 months	Direct and indirect costs
		SHI study population: the high-risk AF group divided into intervention and control groups as close as possible to the CLOSURE-AF protocol	Longitudinal cohort study, retrospective (difference-in-differences)	2015–2021; 1 year before and 4 years after inclusion per individual	
B1	To assess the utility effect of LAAC compared with best medical care	EvaClosure study population: high-risk AF patients divided into intervention and control groups as per the CLOSURE-AF protocol	Longitudinal cohort study, prospective	2018–2025; CLOSURE-AF enrollment starting in 03/2018; obtaining informed consent for prospective and retrospective participation in EvaClosure started in 01/2021, patient individual follow-up of up to 24 months	Health-related quality of life, QALYs
B2	Health economic evaluation of LAAC intervention	EvaClosure study population and SHI study population (cf. module A2)	Cost–utility analysis; Difference-in-differences; Markov model	2 years, 5 years, lifetime horizon	ICER
		CLOSURE-AF trial results			

#### 2.4.1 A1: resource utilization and cost of illness associated with high risk of stroke and bleeding in patients with AF

In the SHI patient database, ICD-10 diagnostic codes will be used to select high-risk AF patients from AF patients without high risk of bleeding and stroke for the cost analysis in module A1 (see section “SHI study population”). A patient's index quarter, regardless of later being allocated to the high-risk group or not, will be the quarter in which the SHI patient inclusion criteria are fulfilled for the first time in the pre-observation year. In addition to these criteria (also using ICD-10 diagnosis codes^6^), patients will be allocated to the high risk of bleeding and stroke group if they have a CHA_2_DS_2_VASc-Score ≥ 2 and one of the following: a HAS-BLED value of ≥3, a diagnostic code for other non-traumatic intracranial hemorrhage, gastrointestinal bleeding, hematuria, bleeding from the respiratory tract, or chronic kidney disease in the observation period. Applicable ICD-10 codes to assess these indications were selected in cooperation with the CLOSURE-AF investigators.

Hierarchically regularized entropy balancing (39, 40), a method for statistical matching, will be used to ensure similar characteristics in terms of age, gender, morbidity (Charlson comorbidity index), and healthcare costs between the high-risk group and the control group without the high risk at baseline.

The two patient groups will be descriptively characterized; location and dispersion measures will be compared with respect to the distribution of appropriate metric variables such as age, morbidity indices, and bleeding risk. Frequencies will be reported for ordinal variables. Differences in mean values for dependent variables, such as resource utilization and direct and indirect costs, will be analyzed by performing a bilateral significance test of independent samples depending on the specific variable distribution in the actual data. The analysis will be conducted by a difference-in-difference design, carried out as two-way fixed effects regression model, alleviating further unobserved structural differences between both groups that may remain after balancing.

The analysis covers SHI data on medical, outpatient hospital and rehabilitation services, inpatient care, drug expenditures, medical aids (e.g., electrostimulation devices and blood pressure monitors), and disability benefits. Indirect costs will be measured by incremental unemployment days and average employee compensation in Germany in the corresponding calendar year, as indicated by the Federal Statistical Office (41).

#### 2.4.2 A2: direct and indirect costs in high-risk AF patients: LAAC procedure vs. best available medical care

To estimate direct and indirect costs, a population with maximum similarity to the EvaClosure study population will be selected from SHI claims data by statistical matching. Age, gender, comorbidity, CHA_2_DS_2_VASc and HAS-BLED scores, and medications (by classification) taken by control group patients will be used for matching of the populations, as observed by the quarter and year in which they and their statistical match meet the inclusion criteria. Matching will be verified using clinical trial data on medication, periprocedural events, (serious) adverse events, and comorbidities detected during follow-up visits of CLOSURE-AF trial patients. In the SHI population, healthcare costs of the LAAC group within a 2- and 5-year time frame will be estimated as the difference between the costs induced by the intervention group and the control group. Indirect costs will be evaluated based on the difference in the average length of incapacity to work documented per group, priced using industry-specific average employee compensation in Germany.

In addition, patient-reported information on health service utilization collected in the EvaClosure study (captured by the FIMA questionnaire as described in the trial data collection section) will be used to validate and supplement the claims data–based cost estimates. Moreover, information on non-medical direct and further indirect costs will be used to calculate societal costs that cannot be determined from SHI claims data. The FIMA questionnaire captures the use of services from general practitioners and specialists, outpatient treatment in hospitals, allied health services, nursing care, partial inpatient care, short-term care, and non-medical services such as housekeeping, assistance from family/friends, and use of other nursing care benefits, over the past three months (35). Similar questions encompass the utilization of inpatient treatment and rehabilitation services, medical devices, and day clinic services in the past 12 months, as well as sociodemographic and socioeconomic data. A validation study based on routine health insurance data (36) showed that resource consumption can be determined validly and reliably by the FIMA questionnaire. Patient-reported health service utilization will then be valued using the valuation rates calculated per observation year in this study, as described by Bock et al. (42) and Muntendorf et al. (43). To cover all components relevant to the target group of EvaClosure patients and to ensure that FIMA data collection is organizationally consistent with the clinical trial visit plan, the questionnaire was slightly modified by the authors of this paper^7^. In particular, the following modifications were made:

- Because patients are surveyed with the FIMA every six months, questions on resource utilization covering a period of 12 months (see above) were modified to cover a period of six months.
- Information on sociodemographic and socioeconomic data as well as on drug utilization is collected in the CLOSURE-AF study. Therefore, questions covering these topics (e.g., on sex, age, marital status, and drug utilization within the last seven days) were waived.
- To estimate the indirect costs, two questions on unemployability (i.e., duration within the last six months and profession) were added.

Using descriptive statistics, measures of location and dispersion will be compared for similarity. Difference-in-differences estimation will be applied to evaluate the incremental costs of the LAAC procedure by comparing the costs of services incurred by the intervention group and the control group. The time frame covers five years, comparing the average costs of both groups before and after the quarter of LAAC or first treatment with one of the pharmaceuticals defined as best medical treatment in the study design section.

#### 2.4.3 B1: the utility effect of LAAC compared with best medical care

The effect of the LAAC intervention will be assessed by QALYs, determined using the EQ-5D-5L questionnaire (cf. trial data collection section). EQ-5D-5L is designed as a generic instrument for use across diseases. Because it is a preference-based index instrument, a utility value can be determined immediately from survey responses (44). The EQ-5D-5L version used in the CLOSURE-AF study has five response options available for each dimension, namely mobility, personal hygiene, general activities, pain/physical complaints, and fear/depression (33), and proved sensitive in recording changes in health-related quality of life and reducing ceiling effects (34, 45).

Assuming no differences in the utilities between groups (LAAC and best medical care) at baseline, QALYs can be calculated by multiplying the mean utility values determined by the EQ-5D-5L at different points in time with the duration of the health conditions. The difference in QALYs between groups will be analyzed by a two-sided, unpaired t-test (or Welch test), providing stronger evidence of a true difference. To determine the total incremental QALYs associated with the intervention, any QALY resulting from reduced age-specific mortality rates will also be considered.

#### 2.4.4 B2: health economic evaluation of LAAC

In module B2, a cost–utility analysis compares LAAC with best medical care. Based on the results of modules A2 and B1, the ICER up to five years after intervention is calculated, with QALYs serving as the main effect measure. The ICER determines the costs to SHI (perspective 1) as well as society (perspective 2) for an additional QALY gained. Depending on the underlying perspective, it will be calculated from direct and indirect costs in SHI claims data, supplemented by an analysis of FIMA data from EvaClosure study patients and QALYs derived from EvaClosure study patients as described in the previous section.

A Markov model adapted from the literature (46) will be employed to capture a lifetime horizon to assess long-term effects on costs and health-related quality of life. The transitions between different health states are linked by probabilities, and the Markov property assumes that the transition probabilities result exclusively from the current state (not from history). The parameters of the initial model (46) will be fine-tuned by SHI claims data estimates to capture probabilities for patients with high-risk AF, with risk estimates evaluated among matched populations of both treatment and control claims data groups. Further relevant parameters depending on the underlying Markov state (costs, utilities) will be derived from SHI claims data analyses and the EvaClosure primary data. Parameters that cannot be sufficiently quantified by one of these data sources may be extracted from the literature or health states may be coarsened. After completion of the CLOSURE-AF trial, clinical study results and comorbidity data of EvaClosure-enrolled patients will be used to adjust the final model.

To compare costs and health effects in different years, future costs and benefits will be discounted by an annual discount rate of 3% for SHI expenditures, indirect costs, and QALYs (47). The influence on the results will be systematically addressed in sensitivity analyses as described in the next section.

**Figure 1:**
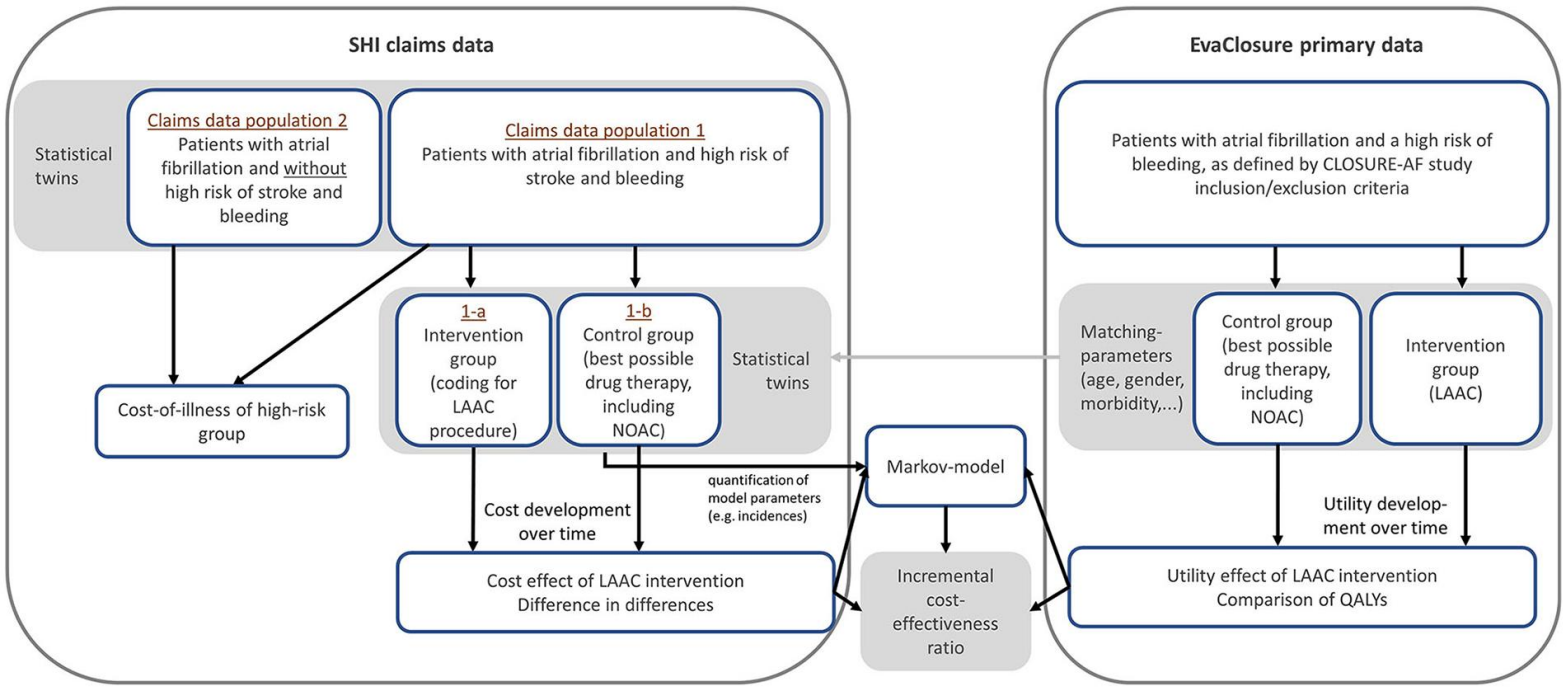
Study populations.

Figure 1 provides an overview of the study design, the main analyses, the two corresponding data sources, and matching criteria to ensure comparability of the SHI- and trial samples.

### 2.5 Sensitivity analyses

Probabilistic sensitivity analyses will address model parameter uncertainty; the effects of each parameter will be analyzed according to its distribution, empirically derived from location and dispersion measures (48). The influence of the discount rate will also be addressed univariately by changing it to 0% and 5% (47). The health economic analysis assumes a linear quality-of-life curve between measurement dates; as recommended by Richardson and Manca (49), we will test the extent to which this assumption influences the results.

After completing the CLOSURE-AF trial, a sensitivity analysis will address the Markov model results by an adjusted parameterization using clinical study results to uncover any influences caused by the EvaClosure sample selection. Because influences of medical–technical progress on the modeling parameters cannot be ruled out in a lifetime horizon, further sensitivity analyses with different transition probabilities will be carried out by a deterministic scenario analysis—factoring in the CLOSURE-AF investigators' clinical expertise—in order to consider these potential effects on the results.

## 3 Discussion

According to available evidence, percutaneous catheter-based LAAC can be considered a potential effective alternative to medical therapies in stroke prevention for AF patients with a high risk of bleeding or contraindications to (N)OACs (50). In the United States, almost 40,000 LAAC procedures were included in the National Cardiovascular Data Registry LAA occlusion registry between 2016 and 2018, of which the majority were performed with favorable outcomes (51), and the European Society of Cardiology recommends considering the procedure for patients with contraindications to (N)OAC therapy in its current guidelines (21). However, distinct clinical superiority and economic benefits of this intervention over the best available drug therapy (standard care) still need to be confirmed, especially in patients at high risk of stroke and bleeding, as addressed by the CLOSURE-AF trial (23). As a health economic substudy, EvaClosure explores LAAC cost-effectiveness from both SHI and societal perspectives when directly compared with the standard drug therapy.

The results from clinical trials that—among other things—consider the occurrence of bleeding or stroke events as well as cardiovascular and all-cause mortality suggest that, for patients with AF, LAAC therapy is not inferior when compared with drug treatment (50). Therefore, we assume that the one-time LAAC procedure costs are compensated over time by the annual savings on drug therapy and fewer complications (e.g., reduced number and duration of inpatient stays due to stroke) (30, 50). The evidence suggests that risks of serious events are lower in LAAC-treated patients compared with the control group, thereby reducing disability and productivity loss in a medium-term time horizon of two years. In the long term (up to five years), we expect a less pronounced increase in outpatient costs, inpatient costs, and overall direct and indirect costs in LAAC–treated patients compared with the control group.

Because of the reduced risks of stroke and bleeding and less need for lifelong medication, we can also assume that LAAC-treated patients report a less deteriorated or even better health-related quality of life in the medium and long term compared with the control group; in other words, assuming no negative effect on life expectancy, there is an expected gain in QALYs from two years after the intervention. If there is a less pronounced increase in costs, this would make LAAC a dominant stroke prevention strategy from both SHI and societal perspectives.

It is a well-known phenomenon that clinical study results may not fully reflect real-world care outside of tightly controlled conditions of clinical trials (52). Our approach of combining trial and real-world SHI data allows us to evaluate the cost-effectiveness of LAAC compared with best medical care [including (N)OAC therapy] but also in contrast to all high-risk AF patients, regardless of their individual (drug) therapy. This largely reflects the situation in the real world, where patients may not fit criteria for standard medication or may switch therapy, making the results particularly applicable to economic decisions in a real-world setting.

Nevertheless, some limitations related to the study design and the data basis must be considered. On the one hand, as with any claims data studies, our data rely on correct documentation of drug therapies, diagnosis codes, and procedures. Errors may have been made in coding, although with the combination of the data used for many of the analyses (drugs, procedure, and codes to evaluate the risk scores), this would be unlikely. On the other hand, the generalizability of the results to other healthcare systems may be limited due to the use of German SHI data. In addition, when using survey or clinical trial data, data/quality issues, for example due to item non-response, may arise and need to be addressed by adequate methods such as multiple imputation. Furthermore, EvaClosure trial participants and SHI claims data population cannot be linked directly at the individual level; to determine the highest possible comparability of costs and data on health-related quality of life, applying statistically matching EvaClosure patients and their SHI claims data counterparts will be crucial to compare primary observational variables among populations, while avoiding potential bias. As multiple data sources [e.g., claims data, survey (trial) data, or literature-based data] will be used, issues such as differences in variable definitions need to be considered. In summary, by integrating clinical and real-world data with multiple health economic evaluation approaches, this study is expected to contribute to evidence-based improvements in the care of patients with AF at high risk for stroke and bleeding.

## Footnotes

^1^CLOSURE-AF is conducted at 42 cardiology centers across Germany, including university hospitals, tertiary referral centers, and high-volume interventional cardiology institutions with established expertise in atrial fibrillation management and structural heart interventions (23, 24).

^2^More detail on the definition is provided in the section ‘Methods’.

^3^For additional information see https://innovationsfonds.g-ba.de/projekte/versorgungsforschung/evaclosure-gesundheitsoekonomische-evaluation-des-perkutanen-verschlusses-des-linken-vorhofohres-bei-patienten-mit-vorhofflimmern-und-hohem-schlaganfall-und-blutungsrisiko-im-vergleich-zur-medikamentoesen-standardtherapie.337 (in German).

^4^Questionnaire for Health-Related Resource Use in an Elderly Population

^5^See section “Outcome measures and analyses” (A2) for details on the modification of the FIMA questionnaire. Modifications were made in consultation with the authors of the original FIMA.

^6^See Supplementary Tables S2, S3 in the Supplementary appendix B and note that drugs are included in calculating the risk scores.

^7^The modified version of the FIMA questionnaire can be made available upon request.
