# Supplementary material for: Health economic evaluation of left atrial appendage closure in patients with atrial fibrillation and at high risk for stroke and bleeding: a study protocol
Source: Front Health Serv. 2026 Jul 1;6:1771683. doi: 10.3389/frhs.2026.1771683 (PMC13368784; doi:10.3389/frhs.2026.1771683)
Supplement: Supplementary file 1 [file Supplementaryfile1.docx]

Supplementary Material

# Appendix

Appendix A: Visit schedule and data collected in the EvaClosure study

Table 1: Visit schedule and data collected for this purpose of the EvaClosure population

| Measureement Designation point | Time point | Collected data | Notes |
| --- | --- | --- | --- |
| Screening-Visit | Day -10-0 | - Inclusion and exclusion criteria - Master data (e.g. age, sex) - Medical history and clinical diagnoses - Comorbidities - Medication | -- |
| Baseline | Day 0 | - Inclusion and exclusion criteria - Master data (Trial arm) - medical history and clinical diagnoses - Medication - Health-related quality of life (EQ-5D-5L) - Use of medical and non-medical services (FIMA) - Accompanying information on the best possible medication - Adverse events | - FIMA only in consecutively enrolled patients of EvaClosure population - Accompanying information on the best possible medication: only control group - Adverse events: only intervention group |
| Intervention | Day 0-7 | - Medication - Accompanying information on the LAAC procedure - Adverse events^2^ | - Accompanying information on the LAAC procedure: only intervention group |
| Pre-Discharge | 1 day after  intervention | - Medication - Adverse events | - Only intervention group |
| V2 | Day 7  (± 3 days) | - Comorbidities - Medication - Adverse events |  |
| V3 | Day 30  (± 7 days) | - Comorbidities - Medication - Adverse events |  |
| V4 | 3 months  (± 1 week) | - Comorbidities - Medication - Adverse events |  |
| V5 | 6 months  (± 2 weeks) | - Comorbidities - Medication - Adverse events - Health-related quality of life (EQ-5D-5L) - Use of medical and non-medical services (FIMA) | FIMA only in patients enrolled in CLOSURE-AF max. 6 months (± 2 weeks) before start of consecutive EvaClosure recruitment |
| V6 | 12 months  (± 1 month) | - Comorbidities - Medication - Adverse events - Health-related quality of life (EQ-5D-5L) - Use of medical and non-medical services (FIMA) | FIMA only in patients enrolled in CLOSURE-AF max. 12 months (± 1 month) prior to start of consecutive EvaClosure recruitment |
| V7 | 18 months  (± 1 month) | - Comorbidities - Medication - Adverse events - Health-related quality of life (EQ-5D-5L) - Use of medical and non-medical services (FIMA) | FIMA only in patients enrolled in CLOSURE-AF max. 18 months (± 1 month) prior to start of consecutive EvaClosure recruitment |
| V8 | 24 months  (± 1 month) | - Comorbidities - Medication - Adverse events - Health-related quality of life (EQ-5D-5L) - Use of medical and non-medical services (FIMA) | FIMA only in patients enrolled in CLOSURE-AF max. 24 months (± 1 month) prior to start of consecutive EvaClosure recruitment |

Appendix B: Criteria for risk-scores and corresponding criteria/ICD in SHI claims data

Table 2 Point values and criteria for determining the CHA_2_DS_2_VASc Score in claims data

| Point value | Criteria in CLOSURE-AF data | Criteria in SHI claims data (Point value if at least one of the ICD codes mentioned is present as an inpatient diagnosis or confirmed outpatient diagnosis or criterion) |
| --- | --- | --- |
| 1 | Heart failure | I50.* - Heart failure I11.0 – Hypertensive heart disease with (congestive) heart failure I13.0 – Hypertensive heart and renal disease with (congestive) heart failure I13.2 – Hypertensive heart and renal disease with both (congestive) heart failure and renal failure |
| 2 | History of ischemic stroke, TIA | I63.*, I64.* - Cerebral infarction G45.09 – Stroke not designated as hemorrhage or infarction Arterial-vertebral syndrome with basilar symptoms G45.89 – Other cerebral transient ischemia and related syndromes I69.2 – Sequelae of other nontraumatic intracranial hemorrhage I69.3 – Sequelae of cerebral infarction |
| 1 | Hypertension | I10.0* - Benign essential hypertension I10.11 – Malignant essential hypertension (with indication of hypertensive crisis) I10.9* - Essential hypertension, unspecified |
| S1 | Diabetes | E10.* - Diabetes mellitus, Type 1 E11.* - Diabetes mellitus, Type 2 E13.* - Other specified diabetes mellitus E14.* - Unspecified diabetes mellitus |
| 1 | Vascular disease | I20.* - Angina pectoris I21.* - Acute myocardial infarction I22.* - Subsequent myocardial infarction I25.* - Chronic ischemic heart disease I70.* - Atherosclerosis I65.* - Occlusion and stenosis of precerebral arteries, not resulting in cerebral infarction I66.* - Occlusion and stenosis of cerebral arteries, not resulting in cerebral infarction I67.* - Other cerebrovascular diseases I71.* - Aortic aneurysm and dissection K55.1 – Chronic vascular disorders of intestine |
| 1 | Age 65 -74 years | 65 ≤ index year – birth year ≤ 74 |
| 2 | Age >=75 years | 75 ≤ index year – birth year |
| 1 | Female sex | Sex = 1 |

Reference: Lip et al. (2010).

Table 3 Point values and criteria for determining the HAS-BLED Score in claims data

| Point value | Criteria in CLOSURE-AF data | Criteria in SHI claims data (Point value if at least one of the ICD codes mentioned is present as an inpatient diagnosis or confirmed outpatient diagnosis or criterion) |
| --- | --- | --- |
| 1 | Uncontrolled hypertension >160mmHg systolic | I11.01, I11.91 - Hypertensive heart disease I13.01, I13.11, I13.21, I13.91 - Hypertensive heart and renal disease I12.01, I12.91 - Hypertensive renal disease I10.01, I10.11, I10.91 - Essential (primary) hypertension (all codes are with indication of hypertensive crisis) |
| 1 | Renal disease^[[1]](#footnote-1)^ | N18.4 or N18.5 – Chronic kidney disease, stages 4 or 5  Z49.1, Z49.2 - Dialysis treatment Z94.0 - Kidney transplant  additional: indicator "extracorporeal blood purification" from record type 100 |
| 1 | Liver disease^[[2]](#footnote-2)^ | K74.3-74.6 - Cirrhosis K70.3 - Alcoholic cirrhosis of liver  R17.0 - Hyperbilirubinaemia with mention of jaundice, not elsewhere classified |
| 1 | Stroke (hemorrhagic and ischemic) | I63.*, I64.* - Cerebral infarction G45.09, G45.89 - Stroke not designated as hemorrhage or infarction arterial-vertebral syndrome with basilar symptoms; other cerebral transient ischemia and related syndromes I60.* - Subarachnoid hemorrhage I61.* - Intracerebral hemorrhage I62.* - Other nontraumatic intracranial hemorrhage  I69.2 - Sequelae of other nontraumatic intracranial hemorrhage I69.3 - Sequelae of cerebral infarction  I69.4 - Sequelae of stroke, not specified as hemorrhage or infarction |
| 1 | History of major bleeding (>=BARC 3) or predisposition to bleeding | I60.* - Subarachnoid hemorrhage I61.* - Intracerebral hemorrhage I62.* - Other nontraumatic intracranial hemorrhage  I69.2 - Sequelae of other nontraumatic intracranial hemorrhage  D62.* - Acute hemorrhagic anemia  Z51.3 - Blood transfusion  K92.2 - Gastrointestinal hemorrhage  H43.1 - Vitreous hemorrhage |
| 1 | Labile INR values (unstable/too high INR values, time in therapeutic range < 60%) | R79.8, R79.9 - Abnormal finding of blood chemistry ^[[3]](#footnote-3)^ |
| 1 | Medication (aspirin, clopidogrel and other antiplatelet drugs, NSAIDs) | Use of at least one of the following agents (presence of one of the following ATC codes)  Antithrombotic agents:  B01AC04 (Clopidogrel), B01AC05 (Ticlopidine), B01AC06 (Acetylsalicylic acid), B01AC08 (Carbasalate calcium), B01AC09 (Epoprostenol), B01AC11 (Iloprost), B01AC13 (Abciximab), B01AC16 (Eptifibatide), B01AC17 (Tirofiban), B01AC21 (Treprostinil), B01AC22 (Prasugrel), B01AC23 (Cilostazol), B01AC24 (Ticagrelor), B01AC25 (Cangrelor), B01AC27 (Selexipag), B01AC30 (Combinations), B01AC56 (Acetylsalicylic acid and Esomeprazol)  NSAR:  M01AA (Butylpyrazolidines), M01AA01 (Phenylbutazone), M01AA02 (Mofebutazone), M01AB01 (Indometacin), M01AB05 (Diclofenac), M01AB09 (Lonazolac), M01AB11 (Acemetacin), M01AB14 (Proglumetacin), M01AB16 (Aceclofenac), M01AB55 (Diclofenac, combinations), M01AC01 (Piroxicam), M01AC05 (Lornoxicam), M01AC06 (Meloxicam), M01AE01 (Ibuprofen), M01AE02 (Naproxen), M01AE03 (Ketoprofen), M01AE09 (Flurbiprofen), M01AE11 (Tiaprofenic acid), M01AE14 (Dexibuprofen), M01AE17 (Dexketoprofen), M01AE51 (Ibuprofen, combinations), M01AE52 (Naproxen and esomeprazole), M01AG (Fenamate), M01AH01 (Celecoxib), M01AH04 (Parecoxib), M01AH05 (Etoricoxib), M01AH06 (Lumiracoxib), M01AX (Other anti-inflammatory and antirheumatic agents, non-steroids), M01AX01 (Nabumetone), M01AX05 (Glucosamine), M01AX24 (Oxaceprol) |
| 1 | Alcohol usage (≥8 drinks/week) | F10.* - Mental and behavioural disorders due to use of alcohol^[[4]](#footnote-4)^ |
| 1 | Age > 65 years | 65 < index year – birth year |

Reference: Pisters et al. (2010).

Literature Cited

Levey, Andrew S.; Stevens, Lesley A.; Schmid, Christopher H.; Zhang, Yaping Lucy; Castro, Alejandro F.; Feldman, Harold I. et al. (2009): A new equation to estimate glomerular filtration rate. In: *Annals of internal medicine* 150 (9), S. 604–612. DOI: 10.7326/0003-4819-150-9-200905050-00006.

Lip, Gregory Y. H.; Nieuwlaat, Robby; Pisters, Ron; Lane, Deirdre A.; Crijns, Harry J. G. M. (2010): Refining clinical risk stratification for predicting stroke and thromboembolism in atrial fibrillation using a novel risk factor-based approach: the euro heart survey on atrial fibrillation. In: *Chest* 137 (2), S. 263–272. DOI: 10.1378/chest.09-1584.

Pisters, Ron; Lane, Deirdre A.; Nieuwlaat, Robby; Vos, Cees B. de; Crijns, Harry J. G. M.; Lip, Gregory Y. H. (2010): A novel user-friendly score (HAS-BLED) to assess 1-year risk of major bleeding in patients with atrial fibrillation: the Euro Heart Survey. In: *Chest* 138 (5), S. 1093–1100. DOI: 10.1378/chest.10-0134.

1. The clinical finding of a creatinine value > 2.26mg/dl / 200µmol/L cannot be depicted exactly. Using the CKD-EPI formula according to Levey et al. 2009 for a white population with an average age of 75, it was estimated that the filtration value eGFR < 30 with the above-mentioned serum creatinine value and thus stages 4 and 5 are relevant. The creatinine value depends, among other things, on stature and gender, which is why, in the case of an admission above stage 4 (N18.4), an additional check is made to determine the extent to which another indication speaks against the admission (e.g. cachexia with ICD-10 R64; E41; B22.2).
   Please kindly note that according to exclusion criteria concerning dialysis or severe chronic kidney disease it might be impossible for patients to fulfil this criterium. [↑](#footnote-ref-1)
2. The clinical findings on the bilirubin value >2x normal value with ALT/AST/AP>3x normal value cannot be mapped. [↑](#footnote-ref-2)
3. The ICD codes are not specific for the INR value. [↑](#footnote-ref-3)
4. The amount of alcohol consumption cannot be distinguished by ICD codes. We assume that the presence of a clinical diagnosis in this regard indicates a conspicuousness and thus the specified minimum number of more than 8 drinks per week. [↑](#footnote-ref-4)
